# Transcriptional Regulation of *PIK3CA* Oncogene by NF-κB in Ovarian Cancer Microenvironment

**DOI:** 10.1371/journal.pone.0001758

**Published:** 2008-03-12

**Authors:** Nuo Yang, Jia Huang, Joel Greshock, Shun Liang, Andrea Barchetti, Kosei Hasegawa, Sarah Kim, Antonis Giannakakis, Chunsheng Li, Anne O'Brien-Jenkins, Dionyssios Katsaros, Ralf Bützow, George Coukos, Lin Zhang

**Affiliations:** 1 Center for Research on Early Detection and Cure of Ovarian Cancer, University of Pennsylvania School of Medicine, Philadelphia, Pennsylvania, United States of America; 2 Department of Obstetrics and Gynecology, University of Pennsylvania School of Medicine, Philadelphia, Pennsylvania, United States of America; 3 Abramson Family Cancer Research Institute, University of Pennsylvania School of Medicine, Philadelphia, Pennsylvania, United States of America; 4 Translational Medicine and Genetics at GlaxoSmithKline, King of Prussia, Pennsylvania, United States of America; 5 Laboratory of Gene Expression, Modern Diagnostic and Therapeutic Methods, Democritus University of Thrace, Alexandroupolis, Greece; 6 Departments of Obstetrics and Gynecology, University of Turin, Turin, Italy; 7 Department of Obstetrics, University of Helsinki, Helsinki, Finland; 8 Department of Gynecology, University of Helsinki, Helsinki, Finland; University of Arkansas, United States of America

## Abstract

*PIK3CA* upregulation, amplification and mutation have been widely reported in ovarian cancers and other tumors, which strongly suggests that *PIK3CA* is a promising therapeutic target. However, to date the mechanisms underlying *PIK3CA* regulation and activation *in vivo* is still unclear. During tumorigenesis, host-tumor interactions may play a critical role in editing the tumor. Here, we report a novel mechanism through which the tumor microenvironment activates the *PIK3CA* oncogene. We show that *PIK3CA* upregulation occurs in non-proliferating tumor regions *in vivo*. We identified and characterized the *PIK3CA* 5′ upstream transcriptional regulatory region and confirmed that *PIK3CA* is transcriptionally regulated through NF-κB pathway. These results offer a new mechanism through which the tumor microenvironment directly activates oncogenic pathways in tumor cells.

## Introduction

Phosphatidylinositol-3′ kinase (PI-3 kinase) is an intracellular transducer with lipid substrate specificity implicated in a wide range of cancer-associated signaling pathways involved in tumor cell metabolism, survival and proliferation [1], [2], [3], [4], [5], [6], [7]. It is recruited and activated by multiple receptor tyrosine kinases and generates second messengers via phosphorylation of membrane inositol lipids at the D3 position [3], [8]. PI-3 kinase was first recognized as putative oncogene because of its ability to bind polyoma middle T antigen [9], [10]. Molecular cloning of PI-3 kinases revealed a large and complex family that contains three classes of multiple subunits and isoforms [3], [8]. However, how each subunit precisely contributes to the progress and maintenance of cancer is largely unknown [3], [5].

The *PIK3CA* gene encodes the catalytic subunit p110-alpha, one of the three catalytic subunit proteins of the class IA PI-3 kinases that are usually activated by growth factor receptor tyrosine kinases. *PIK3CA* was identified as an avian retrovirus-encoded oncogene that transforms chicken embryo fibroblasts [11]. Numerous recent studies indicate that *PIK3CA* and downstream pathways are frequently targeted by genomic amplification, mutation or overexpression in solid tumors including ovarian cancer [12], [13], [14], [15], [16], [17], [18], [19], [20]. Previous studies on the function of *PIK3CA* have predominantly focused on regulatory circuitries within the cancer cell. *In vitro*, *PIK3CA* plays a critical role in cell survival and proliferation [1], [2], [3], [4], [5], [7]. However, to date it remains unknown whether *PIK3CA* assumes diverse roles depending on the state and/or the context in which the tumor cell is found. Furthermore, it remains unclear how such roles of *PIK3CA* might be affected by the tumor milieu.

Dynamic interactions between genetic deregulation in tumor cells and reactive molecular and cellular changes in host cells populating the tumor microenvironment play a critical role in promoting malignant transformation and tumor progression and growth [21], [22]. Following the acquisition of critical genetic alterations, tumor cells are subjected to metabolic/ischemic stress and edit the surrounding microenvironment. In turn, host cells serve to edit the tumor, promoting the selection of tumor cells that benefit from tumor microenvironment influences. Innate and adaptive immune response mechanisms to tumors culminating in inflammation have received significant attention in this context, as inflammation has been shown to promote cancer growth and progression [23], [24], [25]. Tumor-associated leukocytes produce numerous proangiogenic factors promoting tumor vascularization [26], [27], [28], [29]. Furthermore, nuclear factor kappa-B (NF-κB), a critical transcriptional regulator of inflammation, has been shown to play a critical role in inflammation-driven carcinogenesis and to promote tumor growth and progression [25], [30], [31], [32], [33], [34], [35]. It has thus been postulated that the continuous and dynamic cross-talk between tumor cells and host cells ensures the survival of tumor cells and sustains tumor growth [21], [22]. The mutual effects of tumor-host interactions have been characterized with respect to angiogenesis. However, how tumor-host interactions affect directly tumor cells remains partly undetermined.

Epithelial ovarian cancer (EOC), the most common ovarian malignancy, continues to be the leading cause of death among gynecological malignancies [36]. The lack of preventive strategies, early diagnostic methods and effective therapies to treat recurrent ovarian tumors creates a pressing need to understand its pathogenesis and to identify molecular targets for both diagnosis and therapy of EOC at different stage of disease progression [20], [37], [38], [39], [40], [41]. In this study, we examined the expression of *PIK3CA in vivo* and its relationship with the tumor microenvironment in human ovarian cancer. The identification and characterization of the *PIK3CA* 5′ transcriptional regulatory region (TRR) together with functional validation experiments confirmed that *PIK3CA* is transcriptionally regulated by the microenvironment, through inflammation and NF-κB. These results offer a new mechanism through which the tumor microenvironment directly activates oncogenic pathways in tumor cells and promotes tumor growth.

## Materials and Methods

### Patients and Specimens

The specimens used in this study were collected at the University of Pennsylvania and the University of Turin, Italy [42]. Tissues were obtained after patients' written consent under a general tissue collection protocol approved by the institution's Institutional Review Board (IRB) of the University of Pennsylvania and the University of Turin. All tumors were from primary sites, and were collected at the time of debulking surgery from previously untreated patients with stage III and IV ovarian cancer. Samples were snap-frozen immediately and stored at −80°C. Specimens were collected under local Institutional review board approval and processed under procedures approved by the HIPAA act.

### Cell Culture

Cells were cultured in DMEM medium (Invitrogen, Carlsbad, CA) supplemented with 10% fetal bovine serum (FBS, Invitrogen). In some experiments cells were incubated in media enriched with recombinant human TNF-α (50 or 100 ng/ml, BD Bioscience, San Jose, CA) for various times as indicated.

### Total RNA Isolation and Quantitative Real-time RT-PCR

Total RNA was isolated from 100 to 500 mg of frozen tissue or 1×10^6^ cultured cells with TRIzol reagent (Invitrogen). After treatment with RNase-free DNase (Invitrogen), total RNA was reverse-transcribed using Superscript First-Strand Synthesis Kit for RT-PCR (Invitrogen) under conditions defined by the supplier. cDNA was quantified by real-time PCR on the ABI Prism 7900 Sequence Detection System (Applied Biosystems). Human *PIK3CA* forward primer: TCA AAG GAT TGG GCA CTT TT, and reverse primer: GCC TCG ACT TGC CTA TTC AG. PCR was performed using Sybr Green PCR Core reagents (Applied Biosystems, Foster City, CA) according to manufacturer's instructions. PCR amplification of the housekeeping gene GAPDH was performed for each sample as control for sample loading and to allow normalization among samples. A standard curve was constructed with PCR-II TOPO cloning vector (Invitrogen) containing the same inserted fragment and amplified by the real-time PCR.

### Tissue Microarray

The tissue microarray was constructed as described previously [43], [44]. In brief, tumors were embedded in paraffin and 5-µm sections were stained with hematoxylin–eosin to select representative regions for biopsies. Four core tissue biopsies were obtained from each specimen. The presence of tumor tissue on the arrayed samples was verified on hematoxylin–eosin stained section.

### Immunohistochemistry (IHC) and Image Analysis

IHC was performed using the VECTASTAIN ABC Kit as described by the manufacturer (Vector, Burlingame, CA). We used the following primary antibodies (all from BD Pharmingen unless noted otherwise): rabbit anti-human Ki67 (1∶200, DAKO, Carpinteria, CA); rabbit anti-human cytokeratin (1∶200, DAKO); rabbit anti-human p65 (1∶400, Santa Cruz, Santa Cruz, CA); mouse anti-human p110α (1∶250 or 1∶50); rat anti-mouse CD31 (1∶200); mouse anti-human HIF1Α (1∶200); mouse anti-human c-Jun (1∶200); rat anti-mouse CD11b (1∶200). Antibodies were incubated for 2 hrs at room temperature or overnight at 4°C. The immunoreaction was visualized with 3,3′-diaminobenzidine (Vector). Double immunofluorescent staining was performed as previously described [45]. Briefly, sections were sequentially incubated in 5% normal serum; primary antibodies for 2 hrs; and fluorescent labeled secondary antibodies (Vector) for 30 min. Sections were counterstained with DAPI before being inspected under the fluorescent microscope. Images were collected through Cool SNAP Pro color digital camera (Media Cybernetics, Silver Spring, MD) and staining index was analyzed using Image-Pro Plus 4.1 software (Media Cybernetics).

### TUNEL Assay

The ApopTag peroxidase in situ detection kit (Intergen) was used to visualize apoptotic cells *in vivo* and *in vitro*. The procedure was performed according to manufacturer's instruction. Briefly, tumor tissue sections were fixed with 1% paraformaldehyde in PBS, followed by cold ethanol and acetic acid post-fixation. Following incubation with residues of digoxigenin nucleotide and terminal deoxynucleotide transferase for one hr at 37°C, cells were incubated with FITC-labeled anti-digoxigenin antibody.

### Plasmids

The mouse *PIK3CA* cDNA was generously provided by Dr. Roberts (Harvard University). Mammalian expression plasmid pCDNA3 was from Invitrogen. To construct luciferase reporter plasmids, 5′TRRs of the human and mouse *PIK3CA* were amplified from BAC or genomic DNA using Expand High Fidelity PCR System (Roche, Indianapolis, IN) and inserted upstream of the luciferase gene in pGL3-Basic vector (Promega, Madison, WI). Sequences of 5′TRR in all these reporter constructs were verified by DNA sequencing.

### 5′ Rapid Amplification of cDNA Ends (5′RACE)

Total RNA was extracted with the RNeasy Mini RNA kit (Qiagen, Valencia CA) and the 5′ RACE system (Invitrogen) was used. The 5′ RACE PCR product was inserted into the TA cloning system (Invitrogen).

### Plasmid Transfection

Cells were seeded in 6-well plates at 3×10^5^ cells/well and grown overnight to ∼40% confluence prior to transfection. All plasmids were transfected with FuGENE6 transfection reagent (Roche) following the manufacturer's instructions. To select neomycin-resistant cells, 400 µg/ml neomycin (Invitrogen) was applied. For shRNA experiments, cells were transfected with siRNA expressing pLTsuppressor1.0. In vitro experiments indicated that suppression of *PIK3CA* mRNA persisted for up to 30 days. All transfection experiments were done in triplicate and repeated at least twice with different DNA isolates.

### Luciferase Reporter Assay

Cells were seeded in 6-well plates at 3×105 cells/well and grown overnight to 40% confluence prior to transfection. To test the promoter activity of *PIK3CA*, a total of 0.5 µg reporter construct and 0.01 µg pRL-TK internal control (Promega) were used for each transfection. All transfection experiments were done in triplicate and repeated at least twice with different DNA isolates. Forty-eight hours post-transfection, luciferase analysis was performed on Luminoskan Ascent (Thermo-Labsystems, Waltham, MA) using Dual-Luciferase Reporter Assay System (Promega) according to the manufacturers' instructions. For co-transfection experiments, 1 µg of each pCMV-IκBα or pCMV-IκBαM plasmid (CloneTech, Mountain View, CA) was used.

### Electrophoretic Mobility Shift Assay (EMSA)

Nuclear extract from cells was prepared using NE-PER Nuclear and Cytoplasmic Extraction Reagents plus Halt Protease Inhibitor Cocktail Kit (Pierce, Rockford, IL) following the manufacturer's protocols and stored at −80°C until used. Recombinant NFκB (p50) was ordered from Promega. 5′-Biotin-labeled DNA oligos containing the wild-type human *PIK3CA* NFκB binding site (GACGTGGGGGATTTTTCGCGTA), mutated human *PIK3CA* NFκB binding site (GACGTGGGCGATTTTTCGCGTA), scramble human *PIK3CA* NFκB binding site (TCAGATAGACGAGACTTGAGTC), wild-type control NFκB binding site (AGTTGAGGGGACTTTCCCAGG) [35], mutated control NFκB binding site (AGTTGAGGCGACTTTCCCAGG) were synthesized and the two complementary oligos were annealed to obtain the double-stranded probe. EMSA was performed using LightShift Chemiluminescent EMSA Kit (Pierce). The nuclear extract (or p50) and 30 fmol of labeled probe were incubated in the 10× binding buffer plus 1 µg poly(dI–dC) in 20 µl reaction system at room temperature for 20 min. The entire 20 µl binding reaction was loaded on a 7.5% polyacrylamide gel and run at room temperature in 0.25× TBE at 110 V for 1–1.5 h. The electrophoresed binding reaction was then transferred to BrightStar-Plus positively charged Nylon membrane (Ambion, Austin, TX) in Trans-Blot SD Semi-Dry Electrophoretic Transfer Cell (Bio-Rad, Hercules, CA) at 15 V for 30 min and cross-linked in UV Stratalinker 1800 (Stratagene, La Jolla, CA) at auto cross-link level for 1 min. Detection of Biotin-labeled DNA probe was performed strictly following the manufacturer's protocol.

### Chromatin Immunoprecipitation (ChIP)

ChIP assay to detect p65 binding on the human *PIK3CA* promoter was performed using the Upstate ChIP assay kit (Upstate, Charlottesville, VA) following the manufacturer's instruction. Sheared DNA was incubated at 4°C overnight with 2 µg immunoprecipitating rabbit polyclonal IgG antibody to p65 (Santa Cruz) or 2 µg normal rabbit control IgG. Input and chromatin-immunoprecipitated DNA was used as PCR template for detection of p65 binding on the promoter (with primers 5′-GCACCAAGACACTACCTTGAATC-3′ and 5′-CTCTGCAGTCCTTTGACTCACTT-3′) or on the GAPDH promoter (with primers 5′-GACACCATGGGGAAGGTGAA-3′ and 5′-GAGTAGGGACCTCCTGTTTC-3′) using the PCR Core kit (Roche).

### Bioinformatics

A search for potential transcription factor binding sites on the human and mouse *PIK3CA* 5′TRRs were performed using the program Mat Inspector V2.2 at http://www.genomatix.de/online_help/help_matinspector/matinspector_help.html [46].

### Statistics

Statistical analysis was performed using the SPSS statistics software package (SPSS). All results were expressed as mean ± SD, and p<0.05 was used for significance.

## Results

### *PIK3CA* is upregulated in non-proliferating regions in ovarian cancer

**Figure 1:**
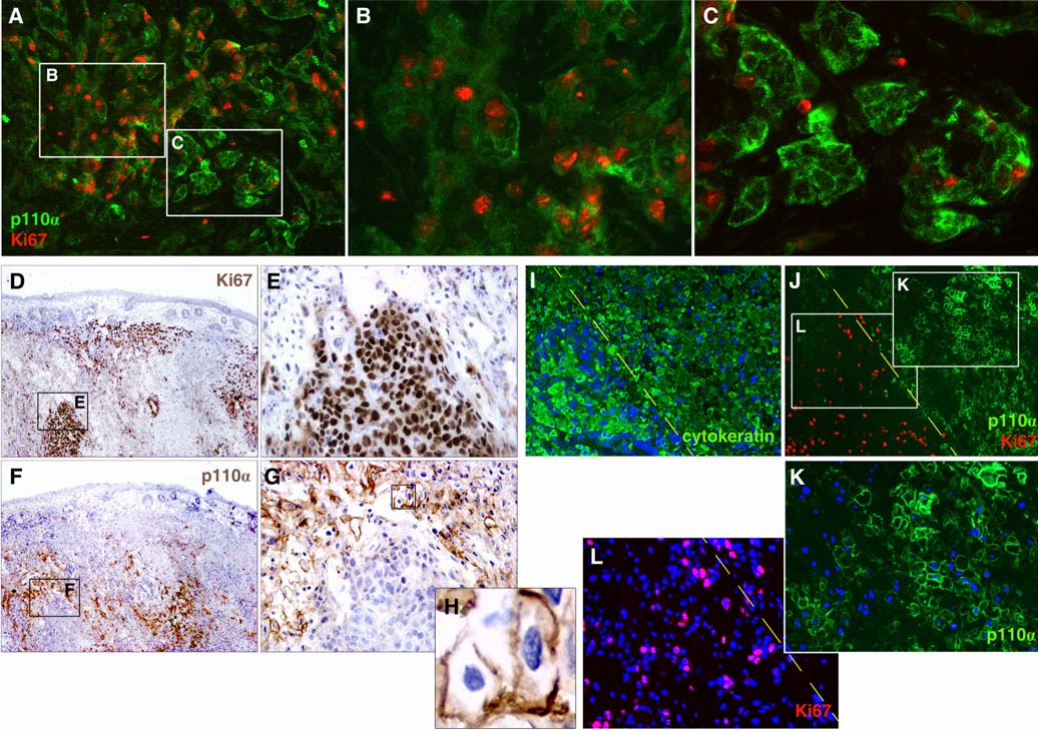
*PIK3CA* is upregulated in non-proliferating tumor cells *in vivo.* A to C. Double immunostaining of p110α (green, FITC) and Ki67 (red, Texas Red) in human ovarian cancer B. High magnification of a region from A with low expression of p110α. p110α is expressed at low levels and localizes to the cytoplasm of Ki67-positive tumor cells. C. High magnification of a region from A with high expression of p110α. p110α is expressed at high levels and localizes to the plasma membrane in Ki67-negative tumor cells. D and E. Immunohistochemical localization of Ki67 allows for clear identification of areas of proliferating and areas of non-proliferating tumor cells *in vivo* in 2008 ovarian xenograft tumors. E. High magnification of area from D showing the boundary between a proliferating and a non-proliferating region. F to H. Strong expression and cell membrane localization of p110α is only found in Ki67-negative areas. F. Section adjacent to D, stained with antibody against human p110α (1:250 dilution). G. High magnification of area from F showing the boundary between proliferating and non-proliferating region. H. High magnification of area from G showing membrane localization of p110α in the non-proliferating region. I. Immunostaining of human cytokeratin identifies tumor cells in proliferating and non-proliferating areas in the 2008 xenograft model. The line traces the boundary between the two areas, as defined by Ki67 staining in adjacent section (see J). Both proliferating and non-proliferating regions are positive for human cytokeratin (FITC, green), indicating tumor cells. Cell nuclei were counterstained with DAPI. J to L. Double p110α and Ki67 immunostaining maps *PIK3CA* activation in proliferating or non-proliferating areas in 2008 xenograft tumors. J. Ki67 (red, Texas Red) and p110α (FITC, green) exhibit reciprocal expression. K and L. High magnification of proliferating region (I) and non-proliferating region (H) from J.

To reveal spatial aspects of *PIK3CA* regulation in tumor *in vivo*, we first examined the expression of p110α in human ovarian cancer specimens. Interestingly, strong p110α expression was mainly detected in groups of non-proliferating, Ki67− tumor cells (Figure 1 A to C). In these cells, p110α was translocated to the cell membrane (Figure 1 C). In contrast, Ki67+ regions exhibited low expression of p110α, which was mainly located in the cytoplasm, while only few Ki67+ cells exhibited p110α at the cell membrane (Figure 1 B). A tissue microarray was used to further validate this result in human ovarian cancer. In 18 of 30 (60%) tumors, strong p110α expression (and membrane localization) was detected mainly in Ki67− tumor regions, while in the other tumors strong membrane p110α expression was either detected both in both Ki67− and Ki67+ tumor cells (5/30, 16.7%); mainly in Ki67+ tumor cells (3/30,10.0%); or it was undetectable (4/30; 13.3%).

**Figure 2:**
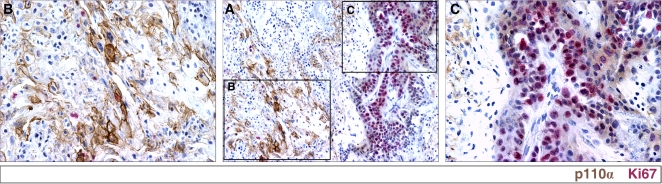
p110α is overexpressed in non-proliferating tumor cells *in vivo.* A. Immunohistochemical staining of p110α using high primary antibody concentration (1:50) reveals low expression of p110α diffusely in the cytoplasm of tumor cells in Ki67-positive (Ki67+) regions. B and C. High magnification of non-proliferating (B) and proliferating (C) regions from A.

**Figure 3:**
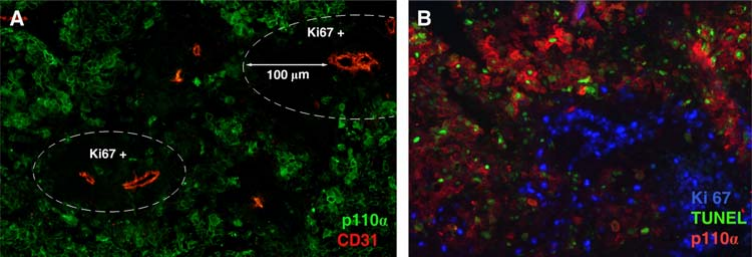
*PIK3CA* is upregulated in undervascularized tumor areas *in vivo.* A. Double immunostaining of CD31 (red, Texas Red) and p110α (green, FITC) in the 2008 xenograft model. Strong p110α staining is mainly detected in tumor regions located distant from capillaries. B. Triple staining of p110α (red, Texas Red), Ki67 (blue, AMCA) and TUNEL (green, FITC) in the 2008 xenograft model.

To further confirm this result, we investigated xenograft tumors generated with the 2008 ovarian cancer cell line. The advantage of this model is that distinct areas of proliferating and resting tumor cells can be clearly identified *in vivo* by Ki67 staining (Figure 1 D and E) [47]. Similarly to the reciprocal expression observed in the human specimens, strong p110α expression was detected in tumor cells only in Ki67−, non-proliferating regions (Figure 1 F to H). In these areas, p110α was mainly localized to the cell membrane (Figure 1 H). Double staining confirmed that areas expressing p110α were in fact populated by tumor cells, which expressed cytokeratin, an epithelial tumor marker (Figure 1 I to K). With increased primary antibody concentration (from 1∶200 to 1∶50), weak p110α expression could also be detected in Ki67+ regions, but p110α localized diffusely to the cytoplasm in these areas (Figure 2). Thus, oncogene *PIK3CA* is significantly upregulated in non-proliferating regions in human ovarian cancer *in vivo*. These non-proliferating regions always lack blood vessel support and contain numerous necrotic/apoptotic cells as well as rich lymphocyte infiltrating (Figure 3).

### Identification and characterization of the human and murine *PIK3CA* promoters

**Figure 4:**
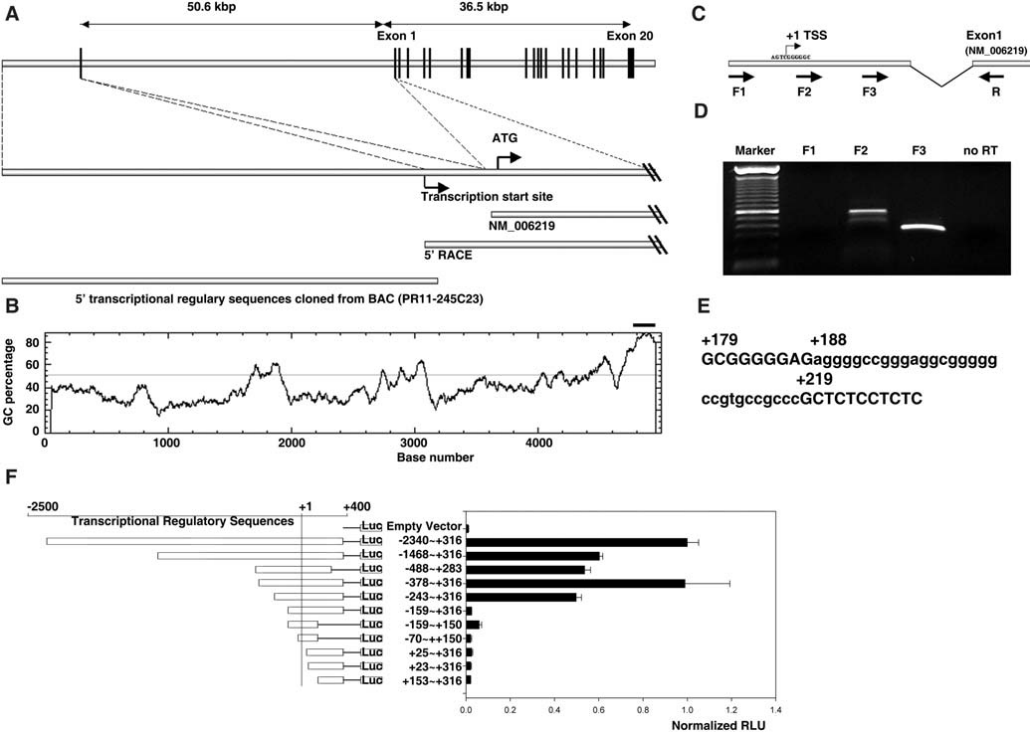
Identification and characterization of the human *PIK3CA* promoters. A. Illustration of the structure of human *PIK3CA* gene and its 5′ upstream regulatory region. B. A region highly rich in GC is found in the *PIK3CA* 5′TRR. C. Illustration of the primers used for mapping RT-PCR of the *PIK3CA* transcriptional start site (SST). D. Results of mapping RT-PCR. There is no band between the forward primer F1 located upstream of the SST and the reverse primer R located on exon 1 of *PIK3CA*. The right size bands could be detected between primer F2 or F3 (both located downstream of SST) and reverse primer R. E. A small splicing variant is found in the 5′UTR of human *PIK3CA* gene, which can also be detected by mapping RT-PCR (primers F2 and R). F. Summarized results of the transcriptional activity of *PIK3CA* TRR fragments.

**Figure 5:**
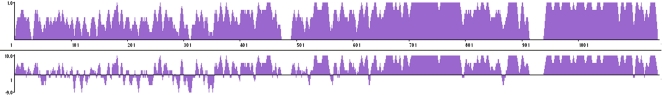
Alignment of human and mouse *PIK3CA* transcriptional regulatory regions.

To better understand the regulation signals contributing to upregulation of *PIK3CA* in cancer, 5′ upstream regulatory sequences of the human *PIK3CA* gene were identified. 5′-rapid amplification of cDNA end (5′RACE) was employed to identify the transcription start site (TSS) of human *PIK3CA* (Figure 4A and B). A 125 bp segment of the 5′-untranslated region (UTR) and 2.3 Kbp of the TRR of human *PIK3CA* were cloned from human bacterial artificial chromosome (BAC, clone number: PR11-245C23). We found that the 5′ upstream regulatory region of human *PIK3CA* is located approximately 50 Kbp upstream of the translation start codon site (Figure 4A). This region is highly rich in GC (Figure 4B). Mapping RT-PCR further confirmed the TSS of *PIK3CA*, and a small splicing variant was identified (Figure 4C to E). The murine *PIK3CA* 5′ upstream regulatory sequence was also cloned by the same method, and was found to be 63.6% identity to the human sequence (Figure 5) and to include a small intron.

To further characterize the transcriptional activity of the promoter region, the entire 2.3 Kbp 5′ TRR or incrementally truncated promoter fragments were subcloned together with a 316 or a 150 bp transcript region into the pGL-basic reporter vector to reveal luciferase expression (Figure 4F). Strong transcriptional activity was localized to the −2,340 to −159 bp region by luciferase assay, while transcriptional activity significantly decreased after −159 bp (Figure 4F).

**Figure 6:**
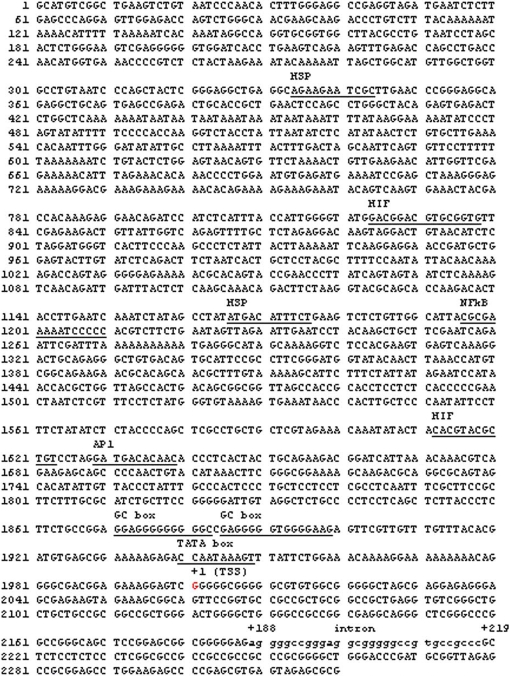
5′ upstream regulatory sequence of the human *PIK3CA* gene.

Next, transcription factor binding sites were predicted on human *PIK3CA* 5′TRR *in silico* by GenomatiX (http://www.genomatix.de/index.html). Binding sites for numerous stress-associated transcription factors were found, including NF-κB; hypoxia-inducible factor (HIF); heat-shock protein (HSP); and activator protein 1 (AP1) (Figure 6). Thus, stress signals mediated by these factors might regulate *PIK3CA* expression in non-proliferating tumor cells in areas of decreased vascularization and increased lymphocyte-infiltrating.

### Expression and localization of candidate transcription factors *in situ*

**Figure 7:**
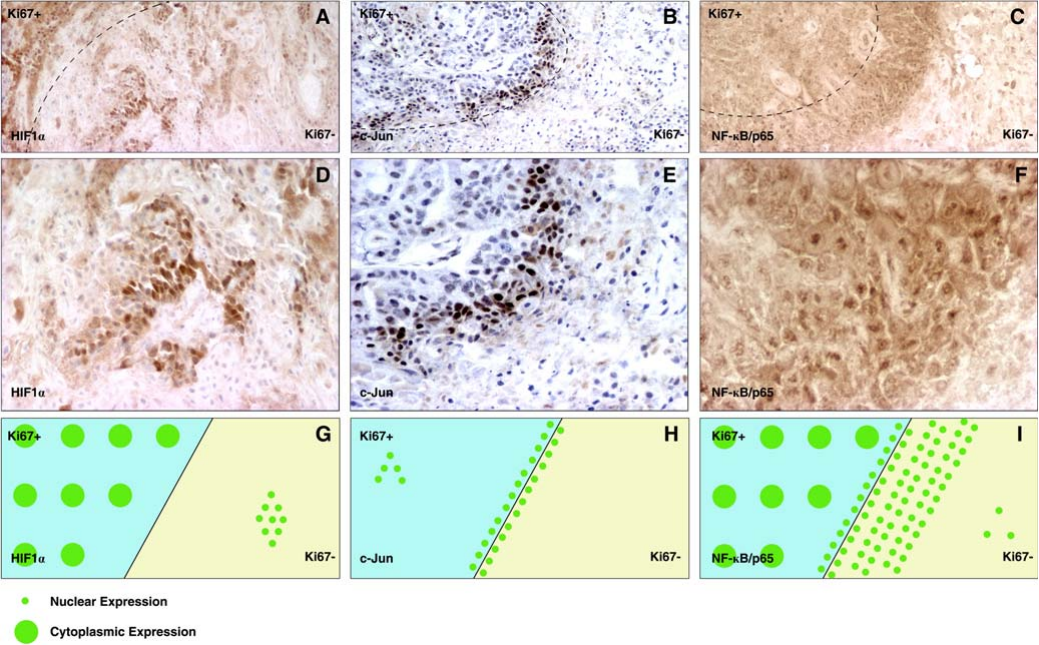
Expression and localization of candidate transcription factors *in situ.* A to C. Immunohistochemical staining of candidate transcription factors HIF1α, c-Jun and NF-κB in 2008 xenografts. The line shows the boundary between the Ki67+ p110α-low, and Ki67− p110α-high regions. D to F. High magnification from A to C, respectively, shows expression and nuclear localization of HIF1Α, c-Jun and NF-κB in 2008 xenografts. G to I. Illustration of the localization of the candidate transcription factors HIF1α, c-Jun and NF-κB in the 2008 xenograft model. Large dots represent cytoplasmic localization, while small dots represent nuclear localization. The line represents the boundary between the Ki67+ p110α-low, and Ki67− p110α-high regions.

The expression and localization of three putative regulatory factors that emerged from the above *in silico* analysis, HIF1α; c-Jun, a member of the AP1 complex; and the p65 subunit of NF-κB, were screened in 2008 xenografts. Because function of these factors requires expression and nuclear translocation, we considered strong expression and nuclear localization indirect evidence of functional activation. Strong nuclear HIF1α expression was seen in patchy cell clusters in Ki67− regions. HIF1α protein was also detected in Ki67+ regions, but it mainly localized to the cytoplasm (Figure 7 A, D and G). c-Jun protein was strictly expressed in the boundaries between Ki67+ and Ki67− regions, where only nuclear staining was detected. Few scattered c-Jun-positive cells could also be detected in Ki67+ regions, but nuclear c-Jun was not seen in Ki67− regions (Figure 7 B, E and H). Strong nuclear localization of NF-κB/p65 protein was seen in the Ki67− regions and in Ki67+ areas adjacent to the Ki67− regions (Figure 7 C, F and I). Interestingly, strongest nuclear NF-κB/p65 was detected adjacent to areas of tissue necrosis (Figure 7 F). This data together with the *PIK3CA* promoter analysis suggest that HIF1α and NF-κB are implicated in the upregulation of *PIK3CA* in Ki67− regions *in vivo*. Consistent with this hypothesis, the hypoxia/HIF pathway has been reported to regulate *PIK3CA* in cancer cells [48]. Since hypoxic regulation of *PIK3CA* has been confirmed, we next focused on the regulation of *PIK3CA* by the NF-κB pathway.

### NF-κB binds to the promoter and upregulates *PIK3CA* expression

**Figure 8:**
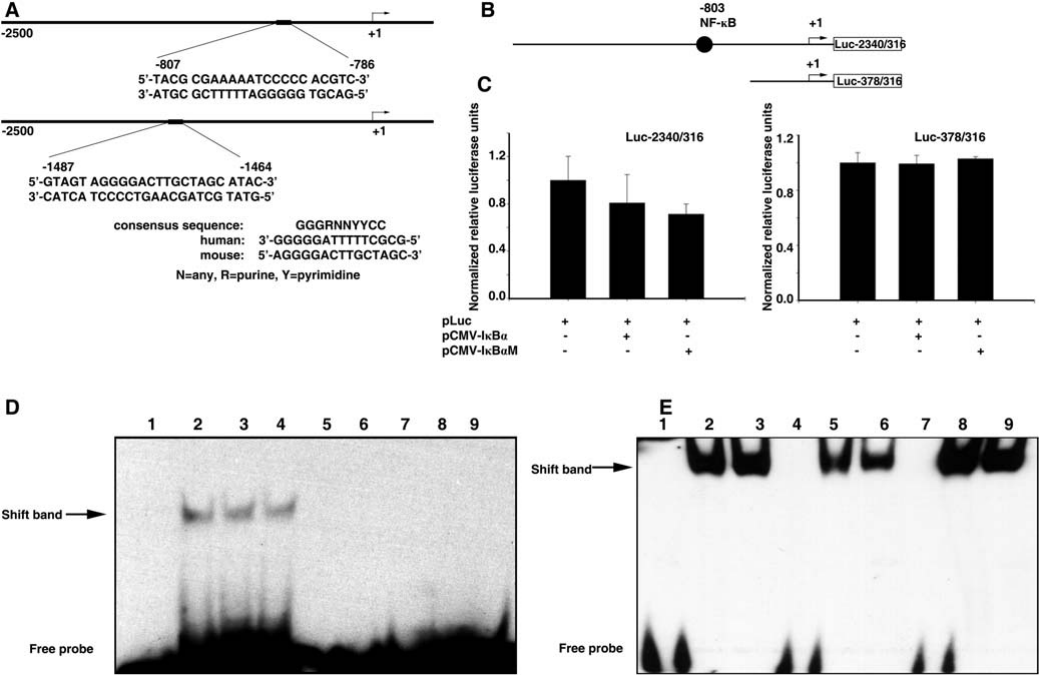
NF-κB binds to the *PIK3CA* promoter and activates its expression. A. Illustration of the predicted NF-κB binding site in human (top) or murine (bottom) *PIK3CA* promoter region. B. Illustration of the constructs comprising luciferase linked to the human *PIK3CA* promoter with (Luc-2340/316) or without the NF-κB binding site (Luc-378/316). C. Summary of luciferase activities of Luc-2340/316 and Luc-378/316 co-transfected with pCMV- IκBα or pCMV- IκBαM. D. Gelshift assay using ovarian cancer cell nuclear extract after TNF-α stimulation. Lane 1, NF-κB binding site of wild-type human *PIK3CA* promoter (wt hu*PIK3CA* NF-κB probe) alone; lanes 2 and 3, wt hu*PIK3CA* NF-κB probe+nuclear extract; lane 4, control NF-κB probe+nuclear extract, lanes 5 and 6, mutated hu*PIK3CA* NF-κB probe+nuclear extract; lane 7, mutated control NF-κB probe+nuclear extract; lanes 8 and 9, scramble hu*PIK3CA* NF-κB probe+nuclear extract. E. Gel shift assay using recombinant NF-κB/p50 protein. Lane 1, wt hu*PIK3CA* NF-κB probe alone; lanes 2 and 3, wt hu*PIK3CA* NF-κB probe+p50; lane 4, mutated hu*PIK3CA* NF-κB probe alone; lanes 5 and 6, mutated hu*PIK3CA* NF-κB probe+p50; lane 7, control NF-κB probe alone; lanes 8 and 9, control NF-κB probe+p50.

Consistent with an important role of NF-κB in regulating *PIK3CA*, we found NF-κB binding site consensus sequences in human and mouse *PIK3CA* 5′TRRs (Figure 8A). The human promoter NF-κB binding site was located at −807 to −786 bp. We tested whether NF-κB regulates the transcriptional activity of human *PIK3CA* promoter *in vitro*. NF-κB activation requires release from its inhibitory subunit IκBα, which allows NF-κB translocation to the nucleus [34], [35]. Transient forced expression of wild-type IκBα or mutant IκBα (IκBαM), both of which bind NF-κB and block its translocation to the nucleus, attenuated the transcriptional activity of *PIK3CA* promoter, as revealed by luciferase assay. Removing the NF-κB binding site from the promoter region abrogated the inhibitory activity of IκBα or IκBαM (Figure 8 B and C).

The predicted NF-κB binding sequence in the human *PIK3CA* promoter was further tested using gel shift assay. Nuclear proteins from 2008 ovarian cancer cell line treated with TNF-α were able to shift (bind) oligonucleotide sequences containing the predicted NF-κB binding site of human *PIK3CA* promoter, but were not able to shift either mutant or mock oligonucleotide sequences (Figure 8 D). In addition, recombinant NF-κB/p50 protein was able to shift the oligonucleotide sequences containing the predicted NF-κB binding sites, confirming their presence in the human *PIK3CA* promoter (Figure 8 E). Finally, the presence of NF-κB binding site in the *PIK3CA* promoter was confirmed by chromosome immunoprecipitation assay (ChIP).

### Inflammation might regulate *PIK3CA* expression via NF-κB pathway

**Figure 9:**
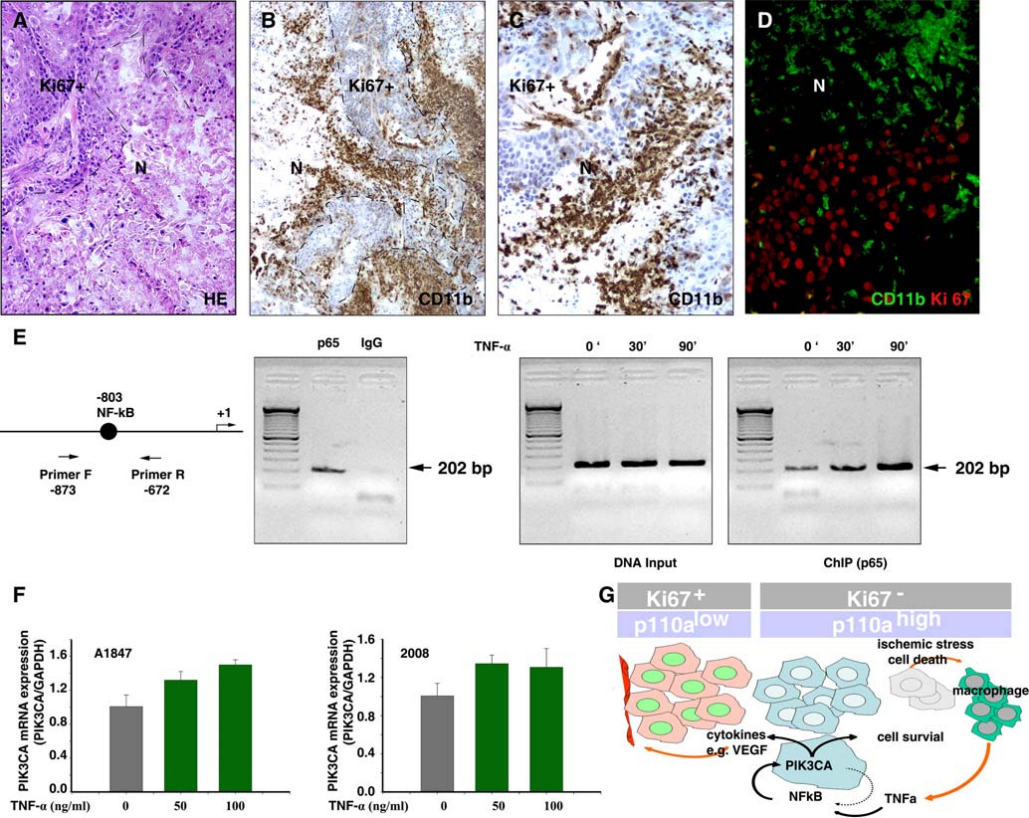
TNF-α regulates *PIK3CA* expression via NF-κB pathway. A. H&E staining of 2008 tumor reveals a prominent area of necrosis (N). B and C. Immunohistochemical staining of murine CD11b reveals macrophage infiltrate in a 2008 xenograft. CD11b+ cells infiltrate tumors in Ki67-negative regions in proximity of necrosis. C. High magnification from B. D. Double immunostaining of CD11b (green, FITC) and Ki67 (red, Texas Red) reveals CD11b+ macrophages mainly in non-proliferating Ki67-negative regions. E. ChIP analysis of NF-κB binding to the endogenous *PIK3CA* promoter. The arrows indicate the positions of the primers flanking −803 NF-κB binding site that were used in the ChIP assays. Cells were treated with TNF-α for 0, 30, or 90 min, and then chromatin protein-DNA complexes were cross-linked using formaldehyde. The purified nucleoprotein complexes were immunoprecipitated with p65 antibody or non-immune IgG and amplified by PCR. F. *PIK3CA* mRNA expression levels after stimulation with pro-inflammatory cytokine TNF-α. G. Illustration of the transcriptional regulation of *PIK3CA* by NF-κB.

During tumor growth, metabolic/ischemic stress induces tumor cell death, recruiting inflammatory cells including macrophages. These release proinflammatory cytokines which activate the NF-κB pathway [25], [33], [34], [35]. We mapped the distribution of necrosis, tumor-infiltrating macrophages and activation of NF-κB in the 2008 xenograft model. Morphologic necrosis was detected predominantly in Ki67− p110α+ regions, often located in their center (Figure 9 A). Brisk infiltration of CD11b+ macrophages, which produce pro-inflammation cytokines such as TNF-α, was found in association with areas of necrosis (Figure 9 B to D).

TNF-α one of major cytokines produced by tumor-associated macrophages, is known to activate NF-κB. Because murine TNF-α is 69% identical and 85% homologous to human TNF-α, and it is known to bind to the human TNF-α receptor with the same affinity and to produce similar biologic effects on human cells as human TNF-α [49], [50], [51], we hypothesized that macrophage-derived TNF-α could be in part responsible for the observed NF-κB activation and *PIK3CA* upregulation in 2008 tumors cells *in vivo*. First, we tested whether TNF-α increases NF-κB complex binding to the *PIK3CA* promoter using the ChIP assay. Antibody to NF-κB/p65 was able to precipitate the *PIK3CA* promoter sequence and this was increased in a time-dependent manner by TNF-α treatment, which increases NF-κB translocation to the nucleus (Figure 9E). Thus, the *PIK3CA* promoter contains a functional NF-κB binding site and can be activated by NF-κB. Lastly, we tested whether TNF-α increased *PIK3CA* mRNA expression in ovarian cancer cell lines *in vitro.* A significant upregulation of *PIK3CA* was found in two ovarian cancer cell lines (Figure 9F). Taken together, our results indicate that inflammation triggered in response to tumor cell death might one of the mechanisms that upregulate *PIK3CA* expression via NF-κB in ovarian cancer cells *in vivo*.

## Discussion

PI-3 kinases are intracellular lipid kinases implicated in the regulation of cell metabolism, survival and proliferation [1], [2], [3], [4], [5]. In this study, we characterized the expression and transcriptional regulation of *PIK3CA* in human ovarian caner *in vivo*. We reported the spatial dissociation between tumor cell proliferation and *PIK3CA* upregulation, and a specialized functional role of *PIK3CA* in non-proliferating tumor cells *in vivo*. The identification and characterization for the first time of the *PIK3CA* 5′ upstream regulatory region confirmed that *PIK3CA* is an important mediator of tumor cell response to stress *in vivo* and enabled the identification of a molecular link between inflammation and tumor growth mediated by NF-κB and p110α.

Based on *in silico* prediction and *in situ* scanning, at least two stress signaling pathways appeared to play important roles in *PIK3CA* regulation in cancer, namely HIF and NF-κB. HIF binding sites were identified in the *PIK3CA* promoter region and strong nuclear HIF staining was detected in the areas where tumor cells upregulate *PIK3CA in vivo*. This is in agreement with recent evidence that *PIK3CA* is upregulated by hypoxia in renal carcinoma cell lines [48]. The second regulatory pathway mediated by NF-κB was characterized in this study. We confirmed NF-κB binding sites on the *PIK3CA* promoter by gel shift; documented binding of NF-κB through ChIP; and showed increased binding of NF-κB to the *PIK3CA* promoter by TNF-α. The use of the 2008 xenograft model enabled us to confirm that activation of NF-κB coincides with p110α upregulation in non-proliferating tumor cells *in vivo*.

NF-κB plays a critical role in inflammation-driven tumor formation, growth, and progression [25], [30], [31], [32], 33, 34, 35. In the classical pathway, NF-κB activation is triggered in response to proinflammatory cytokines that lead to phosphorylation-induced IκB degradation and liberation of NF-κB p50:RelA or p50:c-Rel dimers [25], [35]. NF-κB activation promotes transcription of diverse genes encoding inflammatory cytokines, growth factors and cell adhesion molecules, which can promote tumor growth. Importantly, NF-κB activation may also promote tumor cell survival directly through inhibition of apoptosis or necrosis [34]. The present data offers the evidence that NF-κB directly upregulates *PIK3CA* and provides a novel pathway through which NF-κB activation can promote cancer cell survival during stress. Interestingly, activation of the PI-3 kinase/Akt/mTOR pathway has been previously shown to suppress autophagy and direct apoptosis-resistant tumor cells under stress to necrotic cell death, which results in inflammation and accelerated tumor growth [52].

The present results provide novel *in vivo* evidence to support the concept that tumor-host interactions contribute to oncogene activation and tumor growth. Based on these findings and previously reported evidence, we propose a novel model to interpret the function of *PIK3CA* in solid tumors and its regulation by the tumor microenvironment (Figure 9G). During tumor growth, *PIK3CA* may be expressed at low levels and p110α localizes mainly to the cytoplasm in cancer cells proliferating in proximity to vessels in a metabolically lush microenvironment. This “physiologic” level of *PIK3CA* expression serves as an intracellular mediator maintaining tumor cell proliferation and growth by responding to extracellular growth factors. As tumor cells outgrow nutrient support, cancer cells are subjected to metabolic/ischemic stress ultimately leading to cell death. The paracrine factors released by stressed cells as well as infiltrating-leucocytes can edit the microenvironment towards inflammation. Our data suggest that the inflammatory microenvironment can edit back the tumor cells through activation of the NF-κB/*PIK3CA* pathway, with multifaceted results that promote survival.

In summary, we demonstrated a specialized upregulation of *PIK3CA* oncogene in non-proliferating tumor cells induced by the microenvironment through inflammation. Non-proliferating tumor cells may contribute to resistance to current cancer therapeutics and could be an important source for tumor recurrence. Isoform-specific targeting of *PIK3CA* by small molecule inhibitors or siRNA can significantly block tumor growth and induce apoptosis in human cancer cells [53]. Thus, *PIK3CA*-targeted cancer therapy might be a rational approach to target non-proliferating tumor cells and enhance the effect of chemotherapy or radiation in combinatorial therapy. Furthermore, based on our results, *PIK3CA* targeting might be a rational approach to combine with anti-inflammatory cancer therapy.
